# Effects of Vehicle-Induced Vibrations on the Tensile Performance of Early-Age PVA-ECC

**DOI:** 10.3390/ma12172652

**Published:** 2019-08-21

**Authors:** Xiaodong Zhang, Shuguang Liu, Changwang Yan, Xiaoxiao Wang, Huiwen Wang

**Affiliations:** 1 School of Materials Science and Engineering, Inner Mongolia University of Technology, Hohhot 010051, China; 2 School of Mining and Technology, Inner Mongolia University of Technology, Hohhot 010051, China; 3 School of Civil Engineering, Inner Mongolia University of Technology, Hohhot 010051, China; 4 Beijing Tuowei Times Architectural Design Co., Ltd., Beijing 100020, China

**Keywords:** PVA-ECC, vehicle-induced vibrations, setting periods, tensile performance, grey correlation analysis

## Abstract

Polyvinyl alcohol-engineering cementitious composites (PVA-ECCs) have been widely applied in bridge deck repairing or widening, and a common practice is that a portion of the bridge is left open to traffic while the closed portion is constructed, which exposes the early-age PVA-ECC to vehicle-induced vibrations. However, whether vehicle-induced vibrations affect the performance of early-age PVA-ECC remains unknown. The purpose of this study was to conduct laboratory test programs to investigate to what extent vehicle-induced vibrations soon after installation affects the tensile performance of the PVA-ECC. A self-improved device was used to simulate the vehicle-induced vibrations, and after vibrating with the designed variables, both a uniaxial tensile test and a grey correlation analysis were performed. The results indicated that the effects of vehicle-induced vibrations on the tensile performance of early-age PVA-ECCs were significant, and they generally tended to be negative. In particular, for all of the vibrated PVA-ECC specimens, the most negative effects occurred when vibration occurred during the period between the initial set and the final set. We concluded that although vehicle-induced vibrations during the setting periods had no substantial effects on the inherent strain-hardening characteristics of PVA-ECCs, the effects should not be ignored.

## 1. Introduction

Polyvinyl alcohol-engineering cementitious composites (PVA-ECCs) exhibit remarkable strain-hardening characteristics, and feature outstanding tensile ductility and energy dissipation properties with the strategically designed method of the performance driving design method (PDDA) based on micromechanics [1,2]. Their ultimate tensile strain can steadily stay above 3.0% under a uniaxial tensile load [2,3]. By reducing the brittle nature of concrete, PVA-ECC has opened a new world of possibilities to enhance the safety [4], durability [5,6], and sustainability [7,8] of civil infrastructure.

PVA-ECC has been widely applied in bridge deck pavements [9,10], overlays [11,12], and link slabs [13,14]. However, PVA-ECC does not have obvious economic advantages if it is utilized as the major structural material of a bridge. This material seems more practical and economical if it is used as part of bridge deck repairs. Previous studies have shown that PVA-ECC repairs could effectively deal with bridge deck defects [15] and greatly improve the mechanical performance [13] and durability [6] of a bridge. Nonetheless, the question remains, whether vehicle-induced vibrations affect the tensile performance of PVA-ECC bridge deck repairs. In particular, PVA-ECC is most vulnerable to vibrations during setting periods when vibrations might disrupt its states or physicochemical processes.

The physicochemical processes of PVA-ECCs might be affected by vehicle-induced vibrations during different setting periods. The severe traffic pressure in modern cities increases the possibility that vehicle-induced vibrations will take place adjacent to the setting processes of newly placed PVA-ECC. This will result in the ingredients, such as the hydrates, free water, and fibers in the matrix, being subjected to a continuous disturbance of “inertial force” or a certain amount of acceleration caused by the driving force of vehicle-induced vibrations. Similar to normal concrete, early-age PVA-ECC has rheological properties [16,17], and thus presents different physicochemical states during different setting periods. Therefore, it could be speculated that a series of processes, such as the transportation of water between the pores in the matrix or the interface of the fiber/matrix, the lapping of the structural bonds among hydrates, and the connection of the solid-phase skeleton, might be affected by vehicle-induced vibrations during different setting periods, ranging in time from before the initial set, to during the period between the initial set and the final set, and after the final set.

There are possible effects of vehicle-induced vibrations on PVA-ECC before the initial set. On the one hand, due to good flowability of the PVA-ECC before the initial set [18], the continuous disturbance of vibration during this period might induce a certain degree of bleeding for the PVA-ECC slurry. On the other hand, due to the hydrophilic characteristics of PVA fibers [19], the bleeding process might be accompanied by the floatation of the PVA fibers, resulting in a different distribution density for PVA fibers along the transverse section. Therefore, the tensile performance of PVA-ECC may be affected by these two possible effects before the initial set.

There are possible effects of vehicle-induced vibrations on PVA-ECCs during the period between the initial set and the final set. The periods during the initial set and the final set are the key periods for the growth of hydrates and the formation of microstructures [20]. If vibrated during these periods, the “inertial force” will probably inhibit the agglomeration of C-S-H particles, which is the main process for low-density C-S-H gel transfers to high-density C-S-H gel [21]. Additionally, the vibration will probably damage the bonds of C-S-H gel and result in a certain degree of physical damage or obstruction to the connecting of the solid-skeleton. Moreover, due to the good water absorbability of PVA fibers [22], if vibrated during these periods, the vibration disturbance can promote free water aggregating surround the surface of PVA fibers. This would probably make the water-cement ratio around the interface of the fibers/matrix larger than that of other parts of the matrix, and this then would cause bond reduction for the interface of the fibers/matrix. Furthermore, a large number of ettringite (AFt) precipitates will have formed in the matrix at this stage [23], sufficient free water will make it more conducive to hydroscopic expansion of the AFt at the interface of the fiber/matrix [24], resulting in a larger porosity at the fiber/matrix interface, which would cause further bond reduction for the fibers/matrix interface.

There are possible effects of vehicle-induced vibrations on the PVA-ECC after the final set. After the final set, the remnants of anhydrous cement grains, gradually consumed by hydration, will be enveloped by contiguous, gradually thickening, spherical barrier shells of calcium-silicate hydrate (C-S-H) [25]. During this period, the hydration progress of cement is controlled by the transport of water from capillary pores through the barrier shells toward the interface with anhydrous cement [25]. The continuous disturbance of vibration during this period will probably promote the transport of water from capillary pores through the barrier shells toward the interface with the anhydrous cement, and thus the extra moisture around the anhydrous cement grains will promote their consumption, thereby increasing the degree of hydration and enhancing the strength of the matrix.

As is commonly known, C-S-H gel is the main source for the strength of a cement matrix, and good properties of the cement matrix and the bond of a fiber/matrix interface are necessary for PVA-ECCs to exhibit excellent tensile performance. The above possible effects of vehicle-induced vibrations have a high probability to affect the tensile performance of PVA-ECCs during different setting periods. Therefore, it is necessary to clarify how much vehicle-induced vibrations affect the tensile strength and deformation of PVA-ECCs during different setting periods, ranging in time from before the initial set, to during the initial set and the final set, and after the final set.

For bridge structures, it is necessary to clarify the damage or energy dissipation capacity of the corresponding materials (PVA-ECC or concrete) which were commonly used in the bridge structure and subjected to dynamic loads. A number of correlated studies have been performed with experimental [26,27], methodological [28], and numerical [29,30] investigations, and constructive research progress has been achieved.

Likewise, during the construction or repair of a bridge structure, the effects of vehicle-induced vibrations on early-age bridge deck repairs could not ignored, and they have been examined in many experimental investigations. Thus far, most of the previous studies have focused on how the compressive and bond strengths of “early-age” concrete bridge deck repairs that are affected by vehicle-induced vibrations show no substantial effects [31,32]. In fact, some of the results indicated that if high quality, low slump concrete was used, both the compressive and bond strength appeared to increase slightly [33]. However, “early age” in most related studies was actually the period after the final set of concrete bridge deck repairs. 

Some of the current studies have focused on the effects of vehicle-induced vibrations on the compressive and bond strengths of concrete bridge deck repairs during the setting periods before the final set. Their results showed that if the vibration occurred before the final set, there would be a considerable reduction in the compressive and bond strengths of early-age bridge deck repairs [34]. In particular, studies have shown that, when subjected to vehicle-induced vibrations during the period between the initial set and the final set, concrete bridge deck repairs experienced tensile strength and elastic coefficient losses [35]. Fernandes pointed out that special care must be taken with regard to the tensile strength of concrete repairs, because its reduction can have a negative effect on the usability of a bridge deck, and it can result in a greater deflection [35]. The results of Zhang [36] further confirmed that both visible cracks and serious internal damage of concrete repairs occurred when the concrete was vibrated during the period between the initial set and the final set. He defined this period as the disturbance-sensitive period and inferred that when the concrete was in the disturbance-sensitive period, the bond strength between the cement and the aggregate was incapable of resisting vibration. Therefore, microdefects were induced by vibration that degraded the performance of concrete repairs. However, he did not provide direct evidence for this explanation. Similar conclusions were drawn by Kwan [37] and Ng [38]. Their results showed that if a vibration disturbance started immediately when the concrete repairs were just poured, and when the vibration amplitudes caused by a moving vehicle were greater than 4.5 mm, both the bond and contraflexure strengths were significantly affected (20% reduction). Only one study showed that vehicle-induced vibrations had no substantial effects (less than 10%) on the splitting strength of concrete bridge deck repairs during various setting periods [39].

To summarize, most of the previous studies showed that when vibration occurred after the final set, the effects of vibration had no substantial effects on the strengths of concrete bridge repairs, but when vibration occurred during the period between the initial set and the final set, there was a significant negative impact.

However, prior to our work, to the best of our knowledge, few of the completed studies paid attention to the effects of vehicle-induced vibrations on the tensile performance of early-age PVA-ECC, even though it has bright prospects and it has been widely applied in bridge deck repairs. Among various vibration sources, vehicle-induced vibrations would be the most likely to cause continuous disturbance during the setting periods of PVA-ECCs, and among many methods, the uniaxial tensile test might be the most convenient and effective way to identify the strain-hardening characteristics of PVA-ECC bridge deck repairs. Therefore, in order to determine how much vehicle-induced vibrations affect the tensile performance of early-age PVA-ECCs, we conducted a laboratory test program using a self-improved vibration device that simulated vehicle-induced vibrations. A total of 324 “dog-bone” shaped PVA-ECC specimens with sizes of 330 mm × 60 mm × 15 mm in 36 groups were cast and subjected to vibration tests up to different levels of vibration frequency combined with different lengths of vibration duration at different ages ranging in time from before the initial set, to during the period between the initial set and the final set, and after the final set. After being vibrated with the designed variables, the specimens were tested using a uniaxial tensile test to determine their cracking strengths, ultimate tensile strengths, and strains.

## 2. Materials and Methods 

### 2.1. Materials

The main matrix materials included PO 42.5 R cement, where “PO” represented the “ordinary Portland cement”, classified according to Chinese National Standards GB175-2007 [40], Class-I fly ash, classified according to Chinese National Standards GBJ146-90 [41], and high-quality silica sand. The PO 42.5 R cement was manufactured by Ji Dong Cement in Hohhot, China, and its basic physical indexes and chemical composition were obtained according to Chinese National Standards GB175-2007 [40]; they are listed in Table 1 and Table 2, respectively. The Class-I fly ash with particle sizes in the range from 0.5 μm to 2.0 μm was collected from Ordos Thermal Power Plant, China, and its chemical composition are listed in Table 3. The silica sand with particle sizes in the range from 75 μm to 135 μm was provided by Hohhot Quartz Sand Group Co., Ltd. (Hohhot, China).

The matrix additives that were used to modify the properties of the matrix included 3301E modified polycarboxylate-type superplasticizer (PCSP), JXPT-1206 high-efficiency defoamer (HED), MK-100000S viscosity modifying admixture (VMA), and K-Ⅱ polyvinyl alcohol (PVA) fiber. The PCSP, produced by Dalian Sika Building Materials Co., Ltd., Dalian, China, had a water-reducing efficiency of about 33.0%. The VMA, produced by Shandong Chuangyao Biotechnology Co., Ltd., Jinan, China, was added to enhance the water holding capacity of the matrix. The HED, produced by Beijing Jinliangbo Technology Co., Ltd., Beijing, China, was used to reduce the air content in the matrix. The physical properties of the PVA fiber, provided by Kuraray in Japan, are shown in Table 4. The volume fraction of the PVA fiber was maintained at 2.0% in this investigation. In the mixture design, a water-to-binder ratio of 0.24 was used. The mixture proportions are listed in Table 5.

### 2.2. Methods

#### 2.2.1. Preparation of the Specimens

The mix procedure of the PVA-ECC mixture was as follows. First, the weighed dry composition including the cement, fly ash, silicon sand, and VMA were mixed for two minutes in the cement mortar mixer. Second, the weighted PCSP and HED were dissolved into the weighted water, and then the solution was mixed into the dry mixture and mixed for 6 min in the cement mortar mixer. Finally, the PVA fibers were slowly scattered artificially into the mixture while the mixer was working and then mixed for 6 min after all of the PVA fibers were added.

After mixing, the PVA-ECC slurry was poured into the test mold layer by layer with a scoop-type tool. Thereafter, the newly poured mixture was left to stand in the indoor environment, and it was cured by covering it with plastic sheeting without any disturbance to the designed setting ages. Then the mixture was subjected to the designed vibration variables.

To meet the designed vibration variables, 333 “dog-bone” shaped specimens with sizes of 330 mm × 60 mm × 15 mm were cast in 37 groups (9 in each group), including one group of control specimens that was not subjected to vibration. After being subjected to the vibration variables, the PVA-ECC specimens were cured in a sealable plastic box (there is a layer of water at the bottom of the box and a shelf for placing the specimens, the specimens were not in contact with water, the relative humidity was higher than 90% and the temperature was 20 ± 5 °C). The uniaxial tensile test was performed 28 days after the cement-water contact. To eliminate the variation caused by the individuals, all the specimens were cast by one test operator.

#### 2.2.2. Vibration Device and Variables

**Figure 1:**
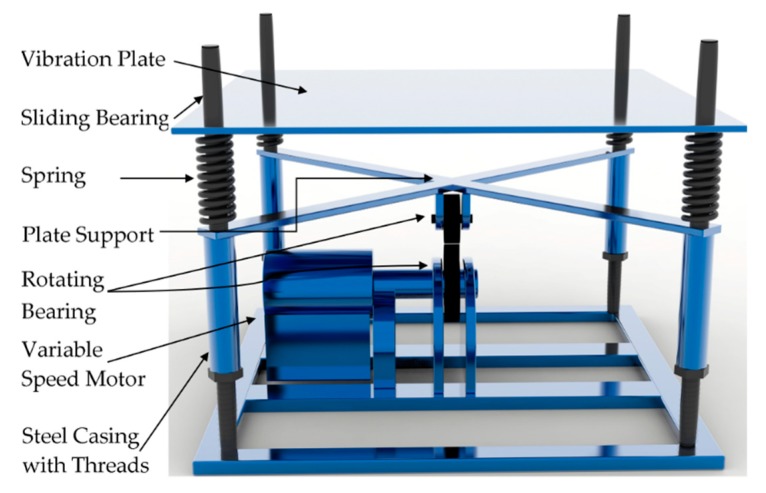
Self-improved vibration device based on cement mortar vibration table.

In order to simulate vehicle-induced vibrations, a vibration device was improved based on the cement mortar vibration table (ZH.DG-80, Hebei Dahong Experimental Instrument, Cangzhou, China), as shown in Figure 1. The driving force of the vibration device was provided by a triphasic variable speed motor. The speed of the motor could be adjusted by the frequency converter, and the vibration frequency of the device would then be converted. To ensure that the vibration model was presented as simple harmonic vibration, two rotating bearings with circular eccentricity were added to the vibration device. 

**Figure 2:**
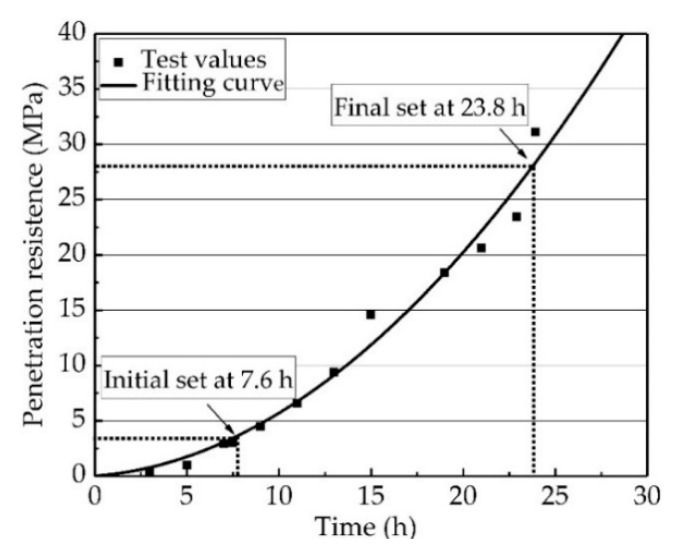
Results from the penetration test showing times of the initial set and the final set.

Before determining the vibration variables, a penetration test needed to be performed to determine the setting time of the PVA-ECC. The setting time of the PVA-ECC was determined based on the penetration resistance test according to JTG E30-2005. The results are shown in Figure 2. From the fitting curve of the time-penetration resistance of the PVA-ECC, the times of the initial set and the final set were obtained, which were 7.6 h (penetration resistance ≤ 3.5 MPa) and 23.8 h (penetration resistance ≤ 28.0 MPa), respectively. 

Three variables were considered to be of the greatest interest for the experimental program: age when vibrated, vibration frequency, and vibration duration. 

The age or time when vibrated was a certain amount of setting time after water-cement contact. This variable was selected to determine the effects of vehicle-induced vibrations on the tensile performance of the PVA-ECC at different ages, ranging in time from before the initial set to during the initial set and the final set and after the final set. The ages when vibrated that we chose in this investigation were 1.5 h, 8. 0 h, 15.0 h, 23.0 h, 36.0 h, and 48.0 h. These six ages ensured that the PVA-ECC would be vibrated throughout the three setting periods.

The vibration frequency was selected to determine the effects of vehicle-induced vibrations on the tensile performance of the PVA-ECC under different levels of dynamics. The vibration frequencies we chose in this investigation were 2.0 Hz, 3.0 Hz, 4.0 Hz, and 5.0 Hz, and the corresponding amplitude was maintained at 5.0 mm.

The vibration duration is the time of maintaining continuous and uninterrupted vibration under the disturbance of one of the four levels of frequency. This variable was selected to explore whether the length of vibration affected the tensile performance of the PVA-ECC. The vibration durations we chose in this investigation were 2.0 h, 5.0 h, 8.0 h, and 11.0 h.

#### 2.2.3. Uniaxial Tensile Test

Uniaxial tensile testing was performed using an electronic universal testing machine (E43.104, MTS Test, Eden Prairie, MN, USA) with a range of 10 kN. The loading rate was maintained as 0.05 mm/s during loading, and the experimental data were collected by a static test analysis system.

**Figure 3:**
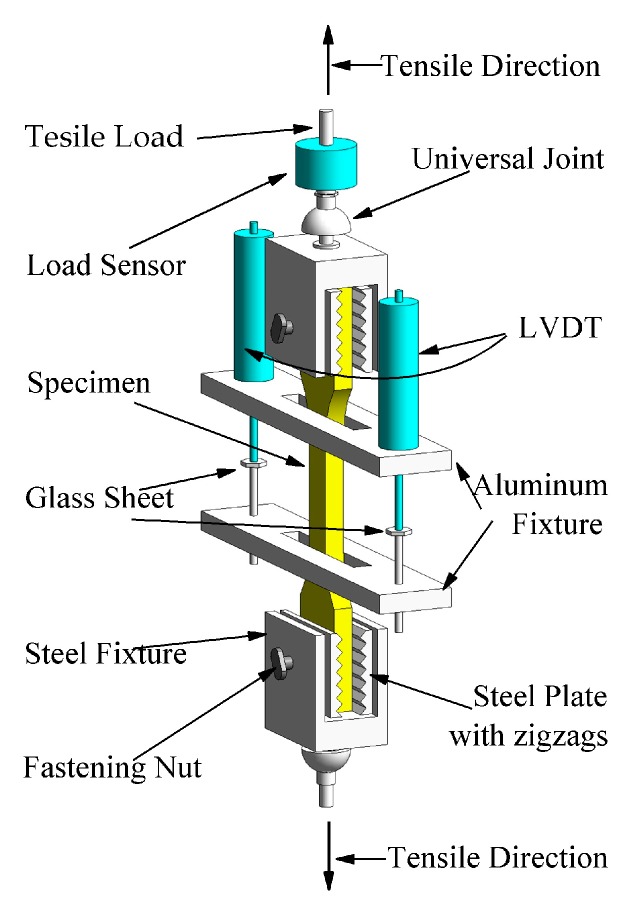
Schematic diagram of the loading device.

The loading device was carefully designed, as shown in Figure 3. To prevent failure occurring at the ends of the specimens caused by uneven extrusion, two steel fixtures were used to clamp the ends of the specimen to produce uniform transmission of the loads. To increase the contact area between the steel fixtures and the sides of the specimen at the ends, four steel plates with zigzag grooves were placed at the contact positions and fastened by four nuts. In order to ensure a uniaxial load and to eliminate eccentric stress for the specimen, two universal spherical hinges were installed at the top of the steel fixture, so that the geometric center line of the steel fixtures could coincide with the axis of the specimen. Before loading, two linear variable differential transformers (LVDTs) were fixed on the sides of the tested specimen with an aluminum fixture, and the measuring distance was maintained at 90 mm in the middle of specimen.

#### 2.2.4. Statistical Analysis

In order to minimize the discreteness of the tested data for the 36 groups of vibrated specimens and the one group of control specimens, the Winsorized mean of the tested stress and strain values of the nine specimens were taken to represent the stress and strain values of the corresponding group. If a certain specimen among the nine specimens had an ultimate tensile strain value that was closest to the Winsorized mean, then its stress-strain curve was taken to represent the stress-strain curve of the corresponding group.

## 3. Results and Discussions

### 3.1. Effects of Age when Vibrated on the Tensile Performance of PVA-ECC

**Figure 4:**
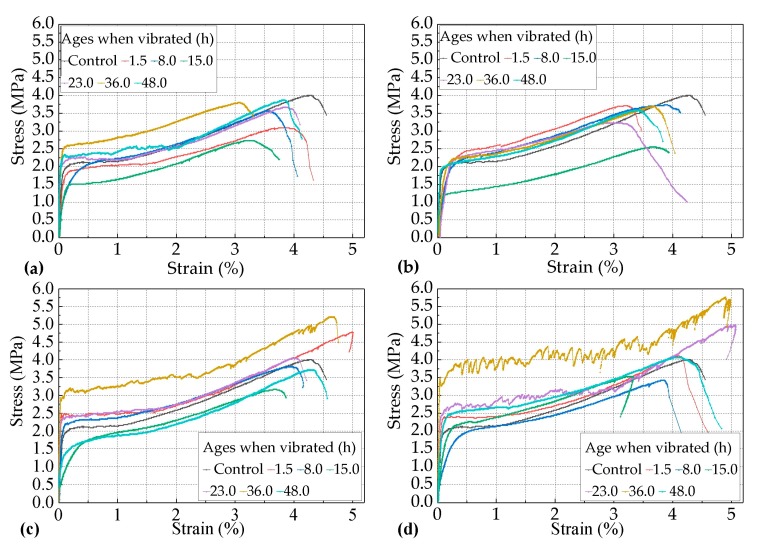
The strain- stress curves of the vibrated PVA-ECC groups and the control group at the ages of 1.5 h, 8.0 h, 15.0 h, 23.0 h, 36.0 h when vibrated subjected to the combination of a constant duration of 5.0 h and different levels of frequency of (**a**) 2.0 Hz, (**b**) 3. 0 Hz, (**c**) 4.0 Hz, and (**d**) 5.0 Hz.

**Figure 5:**
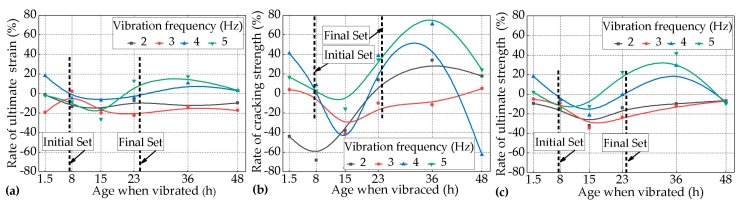
Rates of (**a**) the ultimate tensile strain, (**b**) the cracking strength, and (**c**) the ultimate tensile strength for the vibrated groups over the control group with the increase of the age when vibrated subjected to the combination of different levels of frequency and a constant duration of 5.0 h.

The strain- stress curves of the twenty-four groups of vibrated specimens and the one group of control specimens at different ages when vibrated for 1.5 h, 8.0 h, 15.0 h, 23.0 h, 36.0 h, and 48.0 h, subjected to the combination of a constant duration of 5.0 h and different levels of vibration frequency of 2.0 Hz, 3.0 Hz, 4.0 Hz, and 5.0 Hz, are shown in Figure 4. The rates of cracking strength (σ_cc_), ultimate tensile strength (σ_cu_), and strain (ε_cu_) of the variated specimens over the cracking strength (σ_vc_), ultimate tensile strength (σ_vu_), and strain (ε_vu_) of the control specimens with the increase of ages when vibrated are shown as Figure 5.

#### 3.1.1. Effects of the Age when Vibrated on the Ultimate Tensile Strain of the PVA-ECC

Figure 4 shows that every group of PVA-ECC specimens described in this section exhibited remarkable strain-hardening characteristics and super-high toughness. Their ultimate tensile strain stayed steady above 3.0%, indicating that vehicle-induced vibration had no substantial effects on the strain-hardening characteristics and super-high toughness of the early-age PVA-ECC for this condition. Even so, according to the statistics of the twenty-four groups of vibrated specimens and one group of the control specimen, the probability of ε_vu_ being lower than ε_cu_ was approximately greater than 70%. This indicates that the trend of vehicle-induced vibration on the ultimate tensile strain of early-age PVA-ECC was negative overall.

It can be seen in Figure 5a that for the PVA-ECC specimens subjected to the frequency level of 2.0 Hz, their ε_vu_ values were lower than ε_cu_ throughout the three setting periods, and the most negative effect occurred when the specimens were vibrated at the age of 15.0 h, where ε_vu_ changed by −16.55% over ε_cu_.

For the PVA-ECC specimens subjected to the frequency level of 3.0 Hz, their ε_vu_ changed by 2.10% over ε_cu_ at the age of 8.0 h when the specimens were vibrated, but at the rest of ages when vibrated, the corresponding ε_vu_ changed by −13.29% to −22.38%, and the most negative effect occurred at the age of 23.0 h when the specimens were vibrated, where ε_vu_ changed by −22.38% over ε_cu_.

For the PVA-ECC specimens subjected to the frequency level of 4.0 Hz, their ε_vu_ increased by 18.18% and 10.13% over ε_cu_ at the ages of 1.5 h and 36.0 h when the specimens were vibrated, and their ε_vu_ changed by −3.96% to −6.67% during the period between ages of 1.5 h and 36.0 h when the specimens were vibrated. This indicates that the effects of vehicle-induced vibrations on the ultimate tensile strain of PVA-ECC tended to be slightly negative during the period between the initial set and the final set, and there was a positive effect to some extenet before the initial set or after the final set.

For the PVA-ECC specimens subjected to the frequency level of 5.0 Hz, their ε_vu_ changed approximately linearly from −1.86% to −26.11% over ε_cu_ at relatively lower ages when vibrated (1.5 h to 15.0 h), and the most negative effect occurred at the age of 15.0 h when the specimens were vibrated, where ε_vu_ changed by −26.11% over ε_cu_. By contrast, their ε_vu_ increased within 20% at relatively greater ages (above 23.0 h). These results indicate that the effects of the vehicle-induced vibrations on the ultimate tensile strain of PVA-ECC tended to be negative at relatively earlier ages when vibrated, and it tended to be positive at relatively later ages when vibrated under the frequency level of 5.0 Hz.

To summarize, the effects of vehicle-induced vibrations on the ultimate tensile strain of almost all of the vibrated groups tended to be negative before the final set, while the effects tended to be positive to a certain extent (within 20.0%) for some of the vibrated groups after the final set. In particular, for all of the vibrated groups, the most negative effects occurred during the period between the initial set and the final set, if based on the rate of ultimate tensile strain. For these results, the following explanations can be made.

Before the initial set, vibrations would cause a certain degree of bleeding of the matrix according to normal concrete, and a certain degree of floatation of the PVA fibers that had a hydrophilic property [17], and this resulted in a certain amount of reduction of the ultimate tensile strain. After the final set, the hydration process of the cement was controlled by the diffusion process [25]. The vibrations promoted the transportation of free water from the fiber/cement interface to the surface of the anhydrous cement grains [25], which might have enhanced the strength of the matrix to some extent and at the same time decreased the bond strength of the fiber/cement interface, therefore improving the tensile deformation capacity of the PVA-ECC. However, if vibrated during the period between the initial set and the final set, a series of negative effects might be caused: inhibiting the agglomeration of the C-S-H particles, which was the crucial process to the formation of high-density C-S-H gel [21]; damaging the bonds of C-S-H gels or obstructing them to form a solid-skeleton; and promoting free water aggregation and the convenient hydroscopic expansion of AFt around the interface of the fibers/matrix [23]. These negative effects induced by vibrations might have resulted in a reduction of the bond strength of the interface of fibers/matrix. Therefore, the most negative age when vibrated occurred during the period between the initial set and the final set, if based on the rate of ultimate tensile strain.

#### 3.1.2. Effects of the Age when Vibrated on the Tensile Strength of the PVA-ECC

It can be seen from Figure 5a–c that the impact trends of the vibrations on both the cracking strength and the ultimate tensile strength of vibrated PVA-ECC groups were approximately equal to that of ultimate tensile strain. The differences of the impact degree of vibrations on the strengths were more significant than that of ultimate tensile strain.

It can be seen in Figure 5b that for the vibrated PVA-ECC group subjected to the frequency level of 2.0 Hz, the most negative age when vibrated occurred at age of 8.0 h when vibrated for cracking strength. Except for this, it can be seen in Figure 5b,c that for all of the vibrated groups subjected to the frequency levels of 3.0 Hz to 5.0 Hz, the most negative ages when vibrated occurred at 15.0 h for cracking or ultimate tensile strength. This result indicates that for all of the twenty-four vibrated groups in this section, the most negative age when vibrated for the strength of the PVA-ECC occurred at the periods during the initial set and the final set.

Furthermore, it could be calculated that, for all of the vibrated PVA-ECC groups subjected to the frequency levels of 2.0 Hz to 5.0 Hz, at the most negative ages when vibrated, the cracking strength changed by −68.2%, −41.04%, −75.14%, and −15.61% over the control average, and the ultimate tensile strength changed by −31.84%, −34.58%, −21.39%, and −14.93% over the control average. This result indicates that the impacts degree of the vibrations on the cracking strength of the PVA-ECC were more significant for the combination of different levels of vibration frequency and a constant duration length of 5.0 h at the most negative age when vibrated compared with that of the ultimate tensile strength. Additionally, Figure 5 also shows that the cracking strength was the most sensitive to the variables in this section, and then it followed the ultimate tensile strength and strain. These results were similar to the studies on compressive strength [34], bond strength [35], splitting strength [36] of early-age concrete that was subjected to vibrations during this period.

### 3.2. Effects of the Vibration Duration on the Tensile Performance of the PVA-ECC

**Figure 6:**
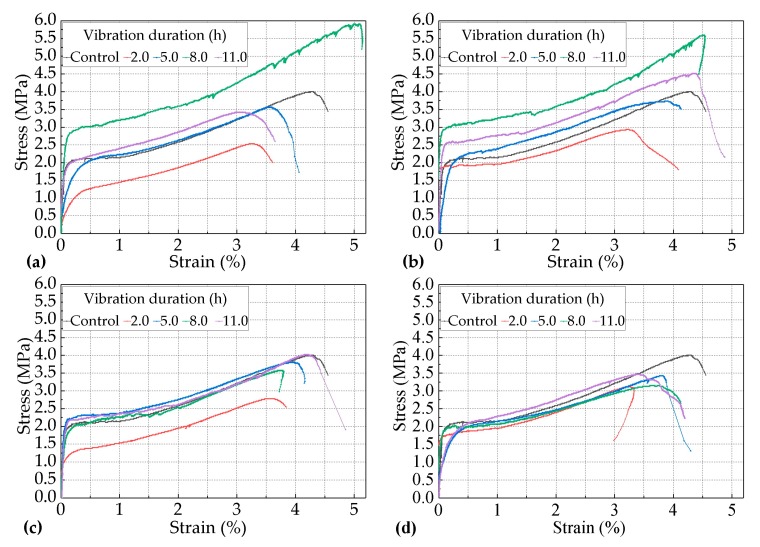
Strain-stress curves of the vibrated PVA-ECC groups subjected to different lengths of vibration duration under the combination of different levels of vibration frequency and the age of 8.0 h when vibrated and, (**a**) 2.0 Hz; (**b**) 3.0 Hz; (**c**) 4.0Hz; (**d**) 5.0 Hz.

**Figure 7:**
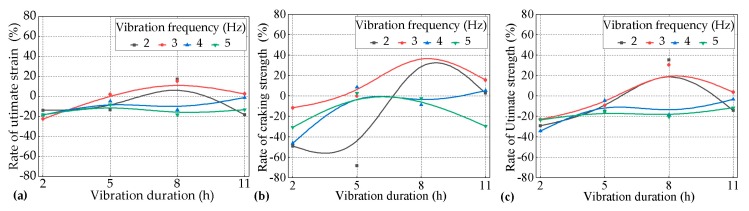
Rates of (**a**) the ultimate tensile strain, (**b**) cracking strength, and (**c**) ultimate tensile strength for the vibrated groups over the control group with the increase of the length of vibration duration under different levels of vibration frequency during the period between the initial set and the final set.

The results in Section 3.1 show that the most negative ages when vibrated for the tensile performance of PVA-ECC occurred during the period between the initial set and the final set. Furthermore, it was necessary to study the effects of the lengths of vibration on the tensile performance of the PVA-ECC at this stage. Therefore, the effects of the vibration duration on the lengths of 2.0 h, 5.0 h, 8.0 h, and 11.0 h on the tensile performance of the PVA-ECC under the combination of different levels of vibration frequency that were within the scope of 2.0–5.0 Hz and the age of 8.0 h when vibrated are investigated in this section. It should be noted that according to the results in Section 3.1, for most of the vibrated groups the most negative age when vibrated occurred at 15.0 h. Here, the age at 8.0 h when vibrated was selected because the corresponding specimens were vibrated only for the period during the initial set and the final set. The strain-stress curves of the sixteen groups of the vibrated PVA-ECC groups and the control group for the variables in this section are shown in Figure 6. The rates of cracking strength, ultimate tensile strength, and strain with the increase of the length of the vibration duration under different levels of vibration frequency at 8.0 h when vibrated over the corresponding control averages are shown in Figure 7.

#### 3.2.1. Effects of the Vibration Duration on the Ultimate Tensile Strain of the PVA-ECC

Figure 6 shows that every group of PVA-ECC specimens described in this section exhibited remarkable strain-hardening characteristics and super-high toughness. Their ultimate tensile strain could stay steady above 3.0% even if the vibrations occurred during the period between the initial set and the final set, with different lengths of vibration duration ranging from 2.0 h–11.0 h. Combined with the results in Section 3.1, it can be further concluded that vehicle-induced vibrations had no substantial effects on the inherent tensile properties of the PVA-ECC within this investigation.

According to Figure 7a, for the vibrated PVA-ECC groups subjected to the frequency level of 2.0 Hz, the rate of the ultimate tensile strain over the control group first increased and then decreased with the increasing of the length of vibration duration. Figure 7a also show that when the vibration duration was 5.0 h, their ε_vu_ decreased by 13.29% over ε_cu_, and when the vibration durations were 2.0 h and 11.0 h, their ε_vu_ decreased by 13.75% and 18.18% over ε_cu_, respectively. However, when the vibration duration was 8.0 h, their ε_vu_ increased by 17.25% over ε_cu_.

For the vibrated PVA-ECC groups subjected to the frequency level of 3.0 Hz, the rate of the ultimate tensile strain over the control group also presented the same trend as that for 2.0 Hz. When the vibration duration was 5.0 h, their ε_vu_ slightly increased by 2.10% over ε_cu_, and when the vibration durations were 8.0 h and 11.0 h, their ε_vu_ increased by 15.38% and 2.56% over ε_cu_, respectively. However, when the vibration duration was 2.0 h, their ε_vu_ decreased by 22.38% over ε_cu_.

When the vibration frequency was 4.0 h or 5.0 h, the curves of the rates of the ultimate tensile strain-vibration durations were relatively smooth for 2.0 Hz or 3.0 Hz, and both of them were below the zero-axis. This result indicates that the effects of the vehicle-induced vibrations on the ultimate tensile strain of the PVA-ECC tended to be negative, but they were not obvious during the period between the initial set and the final set when subjected to relatively higher levels of vibration frequency (4.0 Hz or 5.0 Hz).

To summarize, the above results indicate that the effects of the vibration duration on the ultimate tensile strain of the PVA-ECC tended to be negative overall during the period between the initial set and the final set, but the impact trend and the degree varied for the corresponding lengths of vibration duration and levels of vibration frequency.

#### 3.2.2. Effects of the Vibration Duration on the Tensile Strength of the PVA-ECC

It can be seen in Figure 7 that the impact trend of the vibration duration on the cracking and ultimate tensile strength of the PVA-ECC tended to be coincident with that of the ultimate tensile strain for the period during the initial set and the final set under different levels of frequency, ranging from 2.0 Hz to 5.0 Hz, so this trend will not be repeated here.

The difference was that, except for the individual variation group, the impact degree of the vibration duration on the strengths, including the cracking strength and the ultimate tensile strength, was greater than that of the ultimate tensile stain for the same vibration variables overall. The impact degree of the vibration duration on the cracking strength was greater than that of the ultimate tensile strength. If the defined value of the impact degree was the vibration sensitivity, then combined with Section 3.1, it can be concluded that the cracking strength was the most sensitive to the variables of both the age when vibrated and the vibration duration, and then it followed the ultimate tensile strength and the ultimate tensile strain.

It can also be seen in Figure 7b,c that for all of the vibrated groups of the PVA-ECC, if they were subjected to relatively higher levels of vibration frequency (4.0 Hz or 5.0 Hz), the effects of the vehicle-induced vibrations on their strengths, including the cracking strength and the ultimate tensile strength, tended to be negative but not obvious when they were subjected to different lengths of vibration duration during the period between the initial set and the final set, compared to the relatively lower levels of vibration frequency (2.0 Hz or 3.0 Hz). In addition, a similar trend was obtained to that of the ultimate tensile strain.

### 3.3. Effects of the Vibration Frequency on the Tensile Performance of the PVA-ECC

The results in Section 3.1 and Section 3.2 show that the age when vibrated leading to the most negative effects occurred during the period between the initial set and the final set, and during this period the effects of the vibration duration on the tensile performance of the PVA-ECC were related to the corresponding levels of the frequency. Therefore, it was necessary to study the effects of the vibration frequency on the tensile performance of the PVA-ECC further.

**Figure 8:**
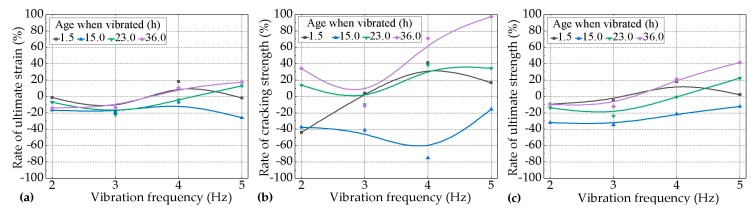
Rates of (**a**) the ultimate tensile strain, (**b**) cracking strength, and (**c**) ultimate tensile strength for the vibrated groups over the control group with the increase of the levels of vibration frequency at different ages with a duration of 5.0 h (Var. 1).

**Figure 9:**
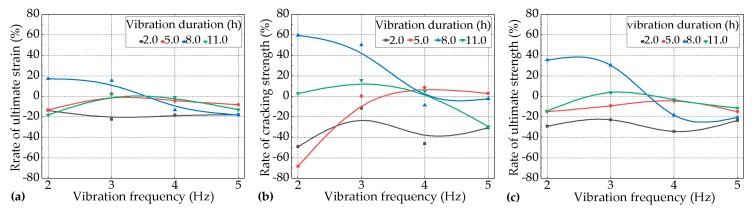
Rates of (**a**) the ultimate tensile strain, (**b**) cracking strength, and (**c**) ultimate tensile strength for the vibrated groups over the control group with the increase of the levels of vibration frequency during the period between the initial set and the final set under different lengths of durations (Var. 2).

The effects of the vibration frequency on the tensile performance of the PVA-ECC were studied under two kinds of variables, as follows: (1) at 1.5 h, 15.0 h, 23.0 h, and 36.0 h, when vibrated with a duration of 5.0 h under different levels of vibration frequency ranging from 2.0 Hz to 5.0 Hz, referred to as Var. 1; (2) with durations of 2.0 h, 5.0 h, 8.0 h, and 11.0 h at 8.0 h when vibrated under different levels of vibration frequency ranging from 2.0 Hz to 5.0 Hz, referred to as Var. 2. The results for Var. 1 and Var. 2 are shown in Figure 8 and Figure 9, respectively.

It can be seen in Figure 8 that for most of the vibrated groups of PVA-ECC for Var. 1, the effects of vehicle-induced vibrations on the tensile performance (cracking strength, ultimate tensile strength and strain) of the PVA-ECC tended to be negative at relatively lower levels of frequency and to be positive at relatively higher levels of frequency, except for the groups that vibrated during the period between the initial set and the final set, which tended to be negative throughout the levels of frequency. 

Furthermore, it can be seen in Figure 9 that for most of the vibrated groups of the PVA-ECC for Var. 2, the effects of vehicle-induced vibrations on the tensile performance of the PVA-ECC tended to be negative throughout the levels of frequency, except for the groups subjected to a duration of 8.0 h, which tended to be positive at relatively lower levels of frequency.

It can be seen in Figure 8 that for the vibrated groups of PVA-ECC for Var. 1, the impact degrees of the vibrations on the cracking strength, ultimate tensile strength, and strain were weaker at relatively lower levels of frequency than those of relatively higher levels of frequency. It can be seen in Figure 9 that for the vibrated groups of the PVA-ECC for Var. 2 the impact degrees of the vibrations on the cracking strength, ultimate tensile strength, and strain were greater at relatively lower levels of frequency than those of relatively higher levels of frequency.

### 3.4. Grey Correlation Analysis of the Factors Affecting the Tensile Performance of the PVA-ECC

Since Professor Deng put forward the grey system theory, grey correlation analysis has become one of the branches that is most widely used and fruitful in grey system theory, and it has been successfully applied to many areas. By comparing the geometric similarity between the curve of the comparative sequence and the reference sequence, the degree of similarity between them can be determined. A closer geometry similarity indicates a greater degree of correlation between the comparative and the reference sequences. The main procedures are shown as follows.

First, the system behavior characteristics $Y_{j}=\left\{y_{j}\left(k\right)\right\}$ were set as the reference sequences, where *k* = 1, 2, ···, n, *j* = 1, 2, ···, *l*. Similarly, the factors that affected the system behavior characteristics $X_{i}=\left\{x_{i}\left(k\right)\right\}$ were set as comparative sequences, where *k*= 1, 2, ···, *n*, *i* =, 2, ···, *m*.

Second, the reference and comparative sequences were initialized as $y_{j}^{\prime }\left(k\right)=y_{j}\left(k\right)/\bar{Y_{j}}$ and $x_{i}^{\prime }\left(k\right)=x_{i}\left(k\right)/\bar{X_{i}}$, respectively. Then the grey correlation coefficients $\xi_{ij}\left(k\right)$ of the reference and comparative sequences at the *k* moment could be expressed as

(1) $$\xi_{ij}\left(k\right)=\frac{\underset{i}{\min}\;\underset{k}{\min}\left|y_{0j}^{\prime }\left(k\right)-x_{i}^{\prime }\left(k\right)\right|+\rho \underset{i}{max}\;\underset{k}{\max}\left|y_{0j}^{\prime }\left(k\right)-x_{i}^{\prime }\left(k\right)\right|}{\left|y_{0j}^{\prime }\left(k\right)-x_{i}^{\prime }\left(k\right)\right|+\rho \underset{i}{max}\;\underset{k}{\max}\left|y_{0j}^{\prime }\left(k\right)-x_{i}^{\prime }\left(k\right)\right|}$$

where ρ∈(0,1) is the distinguishing coefficient, which is used to improve the significance of difference for correlation coefficients, and it is generally taken as 0.5.

Finally, the grey correlation degree $r_{ij}\left(k\right)$ of the comparative sequence $X_{i}$ of the reference sequence $Y_{j}$ could be expressed as

(2) $$\;r_{ij}\left(k\right)=\frac{1}{n}\overset{n}{\underset{k=1}{\sum }}\xi_{ij}\left(k\right)$$

In this study, for all of the 36 groups of PVA-ECC specimens, the corresponding cracking strength, ultimate tensile strength, and strain were taken as reference sequences and recorded successively as $Y_{j}$, *j* = 1, 2, 3. Three vibration factors, the age, duration, and frequency were taken as comparative sequences and recorded successively as $X_{i}$, *i* = 1, 2, 3. The grey correlation degrees of the comparative sequence $X_{i}$ of the reference sequence $Y_{j}$ were calculated as shown in Table 6.

The cracking strength, ultimate tensile strength, and strain of the control specimens were taken as reference sequences, and those of the 24 groups of vibrated specimens for Var. 1 were taken as comparative sequences. The grey correlation degrees were calculated as shown in Table 7. Similarly, the grey correlation degrees for Var. 2 were calculated as shown in Table 8.

In general, it can be seen in Table 6 that the grey correlation degrees of the vibration factors for the tensile performance of the PVA-ECC were greater than 0.6, indicating that the changing curves of the vibration factors were highly correlated to those of the corresponding strengths and strains of PVA-ECC. It also can be seen in Table 6 that the grey correlation degrees of the ages for the tensile performance of PVA-ECC were the lowest compared to those of the durations and frequencies, indicating that the tensile performance of the PVA-ECC was more sensitive to the vibration factor of age when vibrated. 

Further, it can be seen in Table 7 that the grey correlation degrees of the ages at 15.0 h or 23.0 h when vibrated for the tensile performance of the PVA-ECC were the lowest, indicating that the tensile performance of PVA-ECC was most vulnerable to vehicle-induced vibrations during the period between the initial set and the final set, compared to the rest of the setting periods. 

Additionally, it can be seen in Table 8 that the grey correlation degrees of the vibration durations of 8.0 h and 11.0 h for the ultimate tensile strength and strain were relatively lower than that of the vibration durations of 2.0 h and 5.0 h, indicating that the effects of vehicle-induced vibrations were more significant when subjected to relatively longer lengths of vibration during the period between the initial set and the final set.

The results of the grey correlation analysis above were consistent with the results of directive comparison of experimental data in Section 3.3, indicating that the grey correlation analysis was applicable in analyzing the effects of vehicle-induced vibrations on the tensile performance of early-age PVA-ECC in this investigation.

By using this method, the factors affecting the performance of PVA-ECC could be characterized quantitatively with minor calculations, and no excessive requirement for sample size, nor typical distribution rules were needed. Furthermore, even more complete systems could be analyzed to find the major factors, and to quantitatively identify the impact degrees of the factors that affecting the corresponding behaviors.

## 4. Conclusions

According to the results obtained, the following conclusions were drawn.

(1) The effects of vehicle-induced vibrations on the tensile performance, including the cracking strength, ultimate tensile strength, and strain, of almost all of the vibrated PVA-ECC groups that were subjected to a constant vibration duration of 5.0 h tended to be negative before final setting. However, the effects were positive to a certain extent for some of them after the final set. In particular, the most negative ages when vibrated occurred during the period between the initial set and the final set.
(2) During the period between the initial set and the final set, the effects of different vibration durations on the tensile performance of the PVA-ECC tended to be negative overall, but the impact trend and the degree varied for the corresponding lengths and levels of vibration.
(3) The cracking strength was the most sensitive to the variables of both the age when vibrated and the vibration duration in this investigation, and it followed the ultimate tensile strength and the ultimate tensile strain.
(4) The grey correlation analysis was applicable in analyzing the effects of vehicle-induced vibrations on the tensile performance of early-age PVA-ECC.
(5) It can be concluded that although vehicle-induced vibrations during the setting periods had no substantial effects on the inherent strain-hardening characteristics of PVA-ECC, the effects on the tensile performance of PVA-ECC should not be ignored in a practical project. Additionally, the data acquired should be used not only for characterizing the cementitious composite itself in the actual application, but also for developing new models [29] which can explain the measured data.

## Figures and Tables

**Table 1:** Basic physical indexes of PO (ordinary Portland cement) 42.5 R cement.

Initial Set/Final Set (h)	Water Requirement of Normal Consistency (%)	Flexural Strength (MPa)		Compressive Strength (MPa)	
1.95/2.98	26.9	3.0 days	28.0 days	3.0 days	28.0 days
		5.8	8.1	28.9	47.6

**Table 2:** Chemical composition of PO 42.5 R cement (%).

Al_2_O_3_	SiO_2_	CaO	Fe_2_O_3_	MgO	SO_3_	Loss on Ignition
7.19	23.44	55.01	2.96	2.24	2.87	2.86

**Table 3:** Chemical composition of class-I fly ash (%).

SiO_2_	Al_2_O_3_	CaO	Fe_2_O_3_	CO_2_	MgO	SO_3_	K_2_O	Na_2_O	TiO_2_	SrO	Others
40.28	18.15	18.08	8.56	5.18	2.34	2.08	1.76	1.31	0.95	0.73	0.58

**Table 4:** Physical properties of K-Ⅱ polyvinyl alcohol (PVA) fiber.

Fineness (dtex)	Density (g/cm^3^)	Diameter (μm)	Elongation Rate (%)	Tensile Strength (MPa)	Length (mm)	Elastic Modulus (GPa)
15	1.3	40	6	1600	12	40

**Table 5:** Mixture proportions of Polyvinyl alcohol-engineering cementitious composites (PVA-ECCs) (kg/m^3^).

Cement	Fly Ash	Water	Silicon Sand	PCSP	HED	VMA	PVA Fiber
254	1016	304	457	15.24	2.60	0.64	26

**Table 6:** Grey correlation degrees of the vibration factors on the tensile performance of PVA-ECC.

$\mathrm{Grey}\;\mathrm{Correlation}\;\mathrm{Degree}\;r_{ij}$	$\mathrm{Age}\;r_{1j}$	$\mathrm{Duration}\;r_{2j}$	$\mathrm{Frequency}\;r_{3j}$
$\mathrm{Cracking}\;\mathrm{strength}\;r_{i1}$	0.6819	0.9512	0.9607
$\mathrm{Ultimate}\;\mathrm{tensile}\;\mathrm{strength}\;r_{i2}$	0.6674	0.9810	0.9549
$\mathrm{Ultimate}\;\mathrm{tensile}\;\mathrm{strain}\;r_{i3}$	0.6668	0.9800	0.9513

**Table 7:** Grey correlation degrees of the ages when vibrated on the tensile performance of PVA-ECC with a constant of duration of 5.0 h.

$\mathrm{Grey}\;\mathrm{Correlation}\;\mathrm{Degree}\;r_{ij}^{\prime }$	$1.5\;h\;r_{ij}^{\prime }$	$15.0\;h\;r_{2j}^{\prime }$	$23.0\;h\;r_{3j}^{\prime }$	$36.0\;h\;r_{4j}^{\prime }$
$\mathrm{Cracking}\;\mathrm{strength}\;r_{i1}^{\prime }$	0.6514	0.6242	0.7045	0.6539
$\mathrm{Ultimate}\;\mathrm{tensile}\;\mathrm{strength}\;r_{i2}^{\prime }$	0.6620	0.5764	0.5264	0.8298
$\mathrm{Ultimate}\;\mathrm{tensile}\;\mathrm{strain}\;r_{i3}^{\prime }$	0.6514	0.6242	0.7045	0.6539

**Table 8:** Grey correlation degrees of the durations of vibration on the tensile performance of PVA-ECC during the period between the initial set and the final set.

**Grey Correlation Degree** $r_{ij}^{\prime \prime }$	**2.0 h** $r_{ij}^{\prime \prime }$	**5.0 h** $r_{2j}^{\prime \prime }$	**8.0 h** $r_{3j}^{\prime \prime }$	**11.0 h** $r_{4j}^{\prime \prime }$
$\mathrm{Cracking}\;\mathrm{strength}\;r_{i1}^{\prime \prime }$	0.7237	0.6110	0.6463	0.8182
$\mathrm{Ultimate}\;\mathrm{tensile}\;\mathrm{strength}\;r_{i2}^{\prime }$	0.7861	0.8450	0.3946	0.6928
$\mathrm{Ultimate}\;\mathrm{tensile}\;\mathrm{strain}\;r_{i3}^{\prime \prime }$	0.8105	0.6815	0.3676	0.5182
